# Targeted Analysis of HSP70 Isoforms in Human Spermatozoa in the Context of Capacitation and Motility

**DOI:** 10.3390/ijms23126497

**Published:** 2022-06-10

**Authors:** Sarah Grassi, Marie Bisconti, Baptiste Martinet, Vanessa Arcolia, Jean-François Simon, Ruddy Wattiez, Baptiste Leroy, Elise Hennebert

**Affiliations:** 1 Laboratory of Cell Biology, Research Institute for Biosciences, Research Institute for Health Sciences and Technology, University of Mons, Place du Parc 23, 7000 Mons, Belgium; sarah.grassi@alumni.umons.ac.be (S.G.); marie.bisconti@umons.ac.be (M.B.); 2 Evolutionary Biology & Ecology, Université Libre de Bruxelles, Avenue Paul Héger-CP 160/12, 1000 Brussels, Belgium; baptiste.martinet@ulb.be; 3 Clinique de Fertilité Régionale de Mons, CHU Ambroise Paré Hospital, Boulevard Kennedy 2, 7000 Mons, Belgium; vanessa.arcolia@hap.be (V.A.); jean-francois.simon@hap.be (J.-F.S.); 4 Laboratory of Proteomics and Microbiology, CISMa, Research Institute for Biosciences, University of Mons, Place du Parc 23, 7000 Mons, Belgium; ruddy.wattiez@umons.ac.be (R.W.); baptiste.leroy@umons.ac.be (B.L.)

**Keywords:** spermatozoa, HSP70 isoforms, LC–MRM mass spectrometry, capacitation, sperm motility

## Abstract

HSP70s constitute a family of chaperones, some isoforms of which appear to play a role in sperm function. Notably, global proteomic studies analyzing proteins deregulated in asthenozoospermia, a main cause of male infertility characterized by low sperm motility, showed the dysregulation of some HSP70 isoforms. However, to date, no clear trend has been established since the variations in the abundance of HSP70 isoforms differed between studies. The HSPA2 isoform has been reported to play a key role in fertilization, but its dysregulation and possible relocation during capacitation, a maturation process making the spermatozoon capable of fertilizing an oocyte, is debated in the literature. The aim of the present study was to investigate the fate of all sperm HSP70 isoforms during capacitation and in relation to sperm motility. Using Multiple-Reaction Monitoring (MRM) mass spectrometry, we showed that the relative abundance of all detected isoforms was stable between non-capacitated and capacitated spermatozoa. Immunofluorescence using two different antibodies also demonstrated the stability of HSP70 isoform localization during capacitation. We also investigated spermatozoa purified from 20 sperm samples displaying various levels of total and progressive sperm motility. We showed that the abundance of HSP70 isoforms is not correlated to sperm total or progressive motility.

## 1. Introduction

HSP70s, or 70 kDa heat shock proteins, are chaperone proteins essential for the refolding of many newly synthesized or misfolded proteins. They can also allow translocation of proteins across the membrane of organelles, assist in the degradation of unstable proteins, inhibit protein aggregation, dissociate protein aggregates, or even sometimes influence the biological activity of some regulatory proteins [1,2,3,4]. They are found in virtually all organisms in a wide variety of cellular locations [5,6]. In humans, the HSP70 family comprises 13 isoforms that differ according to amino acid composition, tissular expression level and subcellular location [6,7,8]. Some of them are constitutively expressed in cells while others are qualified as “inducible”, i.e., expressed in response to a stress [6,8]. In sperm, up to 12 HSP70 isoforms have been detected. However, this number differs among studies, probably because of the use of different protein extraction buffers and identification methods to investigate the sperm proteome [9,10,11,12,13,14]. In the last ten years, numerous studies have demonstrated the involvement of HSP70 in human sperm function (e.g., [15,16,17,18,19,20]). However, not all these studies identified the involved isoform.

The most studied isoform is HSPA2. Its reduced expression in spermatozoa is linked to altered fertility potential [20,21,22,23]. HSPA2 plays a role in spermatogenesis and fertilization [20,21,24,25]. HSPA2 is first expressed in spermatocytes, in which it supports meiosis, and then in elongating spermatids, in which it is involved in the cytoplasmic extrusion and the remodelling of the sperm plasma membrane to allow its binding to the oocyte zona pellucida [20,21,25]. It has also been demonstrated that, during capacitation, a maturation of the spermatozoa occurring within the female reproductive tract and required for oocyte fertilization, HSPA2 allows the surface relocation of proteins involved in the interaction with the zona pellucida [20,26,27]. The localization of HSPA2 within mature ejaculated spermatozoa is controverted. Indeed, it was shown to be intracellular [20,27], on the plasma membrane surface [28], or intracellular and then relocated on the plasma membrane surface during capacitation [23]. In addition, the protein was described in different regions of the spermatozoa with variations according to the studies (head, acrosomial/ post-acrosomial region, neck, equatorial segment, tail, or connecting piece) and some authors stated that the protein distribution varied following capacitation while others showed the opposite [20,23,26,27,29,30]. In proteomic studies, HSPA2 abundance was found to vary following capacitation [31] or acrosome reaction [32].

Some HSP70 isoforms have been shown to be involved in human sperm motility. Several studies that compared the proteome of asthenozoospermic (i.e., with a low percentage of motile spermatozoa) and normozoospermic samples identified variations in the abundance of different isoforms [15,17,33,34,35,36,37,38]. However, high variability was observed in the results reported in these studies, with opposite variations observed for the same isoform (Table S1). In addition, comparing the proteome of two sperm subpopulations (motile vs. non-motile) of normozoospermic samples, Amaral et al. [15] measured a lower abundance of HSPA4L and HSPA9 in the non-motile subpopulation. Using immunofluorescence and Western blot analyses, Liu et al. [18,39] showed that HSPA4L was less expressed in spermatozoa from asthenozoospermic and teratozoospermic (i.e., with less than 4% of spermatozoa with normal morphology) samples than in normozoospermic samples.

Finally, variations in the abundance of HSP70 isoforms have also been reported in some studies focused on human sperm cryopreservation. Bogle et al. [40] observed a decrease in HSPA4L abundance after the addition of a protein-free cryoprotectant to the sperm samples. Comparing the proteome of fresh and cryopreserved (using cryostraws and cryovials) spermatozoa, Li et al. [41] observed that both cryopreservation methods induced a decrease in the level of different HSP70 isoforms. However, in other proteomic studies, no variation in the abundance of HSP70 isoforms was observed after vitrification [42] or cryopreservation using a glycerol-yolk freezing medium [43].

The studies cited above demonstrate the importance of HSP70 chaperone proteins in human spermatozoa as well as the necessity of distinguishing the involved isoform(s) in the investigated process. However, some discrepancies exist between different studies regarding the variation in abundance and localization of specific isoforms. In the present study, we developed a method for the targeted analysis of each individual HSP70 isoform, by Multiple-Reaction Monitoring (MRM), a very robust and sensitive mass spectrometry method [44,45]. First, we investigated the abundance of each isoform in the context of capacitation. Indeed, although global proteomic changes have been investigated during this process in human and other mammalian species [31,32,46,47,48,49,50,51,52,53], HSP70 isoforms have not been specifically targeted. In addition, we also investigated the localization of HSP70 in the same experimental conditions using immunofluorescence with two antibodies targeting distinct isoforms. Finally, we also investigated the abundance of each HSP70 isoform in a cohort of 20 patients, whose semen samples varied in the percentage of motile spermatozoa, to reconcile results obtained in global proteomic studies (Table S1), [15,17,34,35,36,37].

## 2. Results

### 2.1. Parameters of Sperm Samples Included in This Study

Twenty-two samples were included in this study. Their parameters, information from the donors and the experiment(s) for which they were used are presented in Table S2. For the comparison of capacitated and non-capacitated samples, only normozoospermic samples, as described in Materials and Methods section, were used. Some of these samples had <4% normal forms but we considered this to have a limited effect as the comparison between capacitated and non-capacitated spermatozoa was performed within the same sample. For the investigation on the correlation between HSP70 abundance and sperm motility, we used 20 samples presenting various sperm motility, some of them having <4% normal forms. Noteworthy, three of the donors were obese (BMI > 30 Kg/m^2^) (Table S2).

### 2.2. Evaluation of Sperm Capacitation

**Figure 1:**
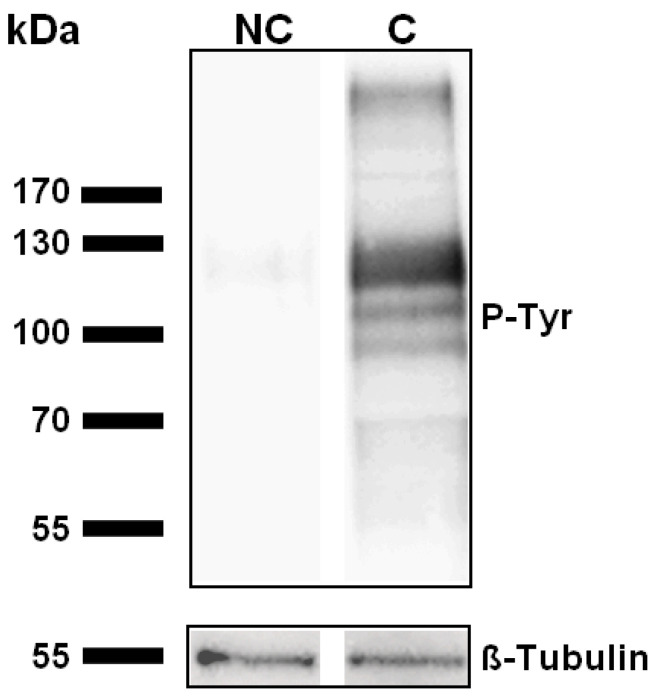
Assessment of sperm capacitation through protein tyrosine phosphorylation. Purified human spermatozoa were incubated (C) or not (NC) in a capacitating medium for 4 h. Proteins were then extracted and submitted to an immunoblot using anti-phosphotyrosine antibody (clone 4G10, Sigma-Aldrich). β-Tubulin was used as an internal reference. Representative results of N = 6 experiments.

The efficiency of capacitation was assessed by analyzing the level of tyrosine-phosphorylated protein in Western blot, tyrosine phosphorylation being considered as a hallmark for sperm capacitation [54]. No labelling was observed in non-capacitated samples while, after incubation for 4 h in the capacitating medium, a labelling was observed at the level of three main bands comprised between 90 and 120 kDa, as observed in other studies [55,56] (Figure 1).

In order to ensure that non-capacitated and capacitated samples could be compared in the subsequent analyses, we analyzed sperm vitality and motility in both conditions (Table 1 and Table S5). Mean values for these parameters were similar to those obtained in other studies using the same capacitation medium [57,58]. No significant difference was observed for the progressive and total motility between non-capacitated and capacitated spermatozoa. As for vitality, although a statistical difference was observed (*p* = 0.043), the percentage of viable spermatozoa was closed to 90% in each case (Table 1).

### 2.3. Targeted Quantification of HSP70 Isoforms Using Multiple-Reaction Monitoring (MRM)–Mass Spectrometry

Based on the alignment obtained with the protein sequences of human HSP70 isoforms, tryptic peptides specific to each isoform were identified and searched in a sperm proteome obtained as detailed in the Materials and Methods section. No specific peptide for isoforms HSPA6, HSPA7, HSPA12A, HSPA12B, HSPA13 and HSPA14 was found in the proteome. Therefore, these isoforms were not included in our study. Moreover, no peptide allowed to discriminate HSPA1A and HSPA1B, which were therefore considered here as a unique polypeptide. Following MRM optimization, we kept a minimum of two peptides to quantify each of the eight considered isoforms (Figure S1).

MRM relative quantification was performed on non-capacitated and capacitated spermatozoa from six normozoospermic individuals. Raw data are available in Table S6. The intensity of each isoform, normalized to the intensity of aconitate hydratase (selected as an internal control), was used to calculate a fold change between non-capacitated and capacitated spermatozoa. According to the isoform, the fold change was comprised between 1.03 and 1.09 (Table 2). Except for HSPA5, no significant difference was observed for HSP70 isoform relative abundances between non-capacitated and capacitated spermatozoa (Table 2). However, although statistically significant (*p* = 0.019), the mean fold change measured for HSPA5 (1.08) is close to 1 and therefore does not reflect a change in HSPA5 abundance between non-capacitated and capacitated spermatozoa.

MRM quantification was also performed to investigate HSP70 abundance in spermatozoa purified from 20 sperm samples presenting various total and progressive sperm motilities (Tables S2 and S7). In that case, we used Tektin 2 as an internal control, as described in the Materials and Methods section. No significant correlation was observed between the abundance of each HSP70 isoform and the percentage of motile and progressive spermatozoa measured in the raw semen samples (Table 3).

Among the different samples obtained for this study, we selected some which belonged to distinct groups defined as follows: (1) astheno-teratozoospermic samples (N = 3), with sperm count > 16 × 10^6^/mL, total motility < 42%, progressive motility < 30% and <4% normal forms, (2) normozoospermic samples (N = 7), with sperm count > 16 × 10^6^/mL, total motility ≥ 50%, progressive motility ≥ 40% and ≥4% normal forms, and (3) teratozoospermic samples (N = 3), with sperm count > 16 × 10^6^/mL, total motility ≥ 50%, progressive motility ≥ 40% and <4% normal forms (Table S2). As expected due to the selection of parameters defining the groups, total and progressive motility were significantly different between the astheno-teratozoospermic group and the two other groups (*p*-values < 0.05), but no significant difference was observed between the normozoospermic group and the teratozoospermic group for both parameters (Tables S8 and S9). However, no significant difference was measured between the three groups regarding the abundance of the different HSP70 isoforms (Table 4).

### 2.4. HSP70 Immunolocalization in Capacitated and Non-Capacitated Spermatozoa

**Figure 2:**
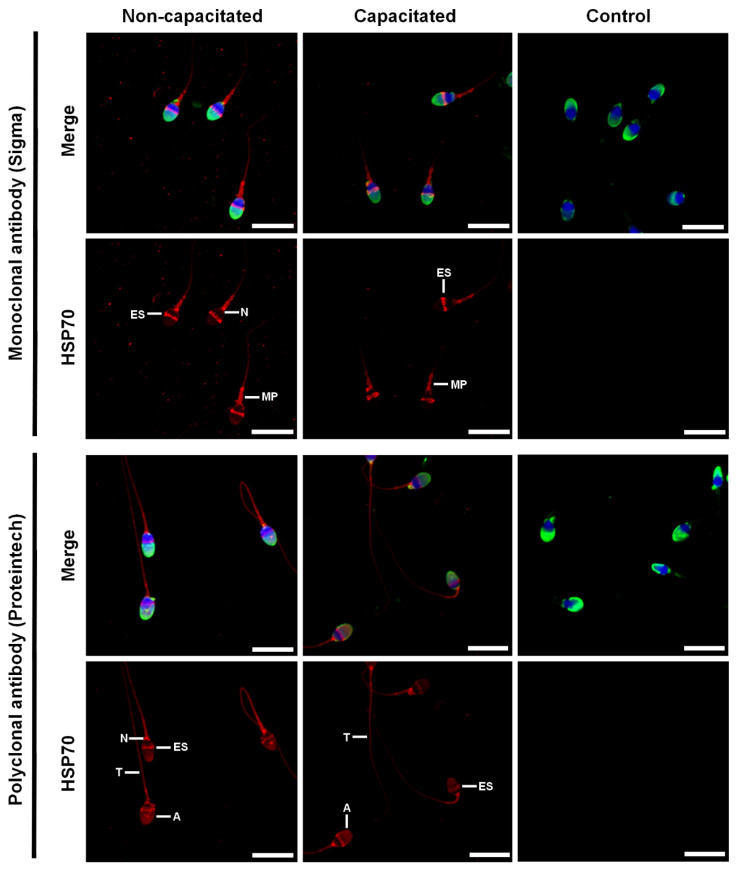
Localization of HSP70 in fixed and permeabilized human spermatozoa. Purified human spermatozoa were incubated or not in a capacitating medium for 4 h. They were then fixed with 4% paraformaldehyde, permeabilized with 0.3% Triton-X-100 and stained with monoclonal (Sigma, H5147) or polyclonal (Proteintech, 10995-1-AP) anti-HSP70 antibodies. Negative controls were performed by incubating non-capacitated spermatozoa without primary antibodies. Red: Hsp70, Blue: DAPI staining of the nucleus, Green: PSA-FITC staining of the acrosome. Scale bar: 10 μm. Images are maximum-intensity projections (MaxIP) obtained from z stack images using Nikon NIS Elements software. Representative results of N = 4 experiments. A: acrosome, ES: equatorial segment, MP: mid-piece, N: neck, and T: tail.

**Figure 3:**
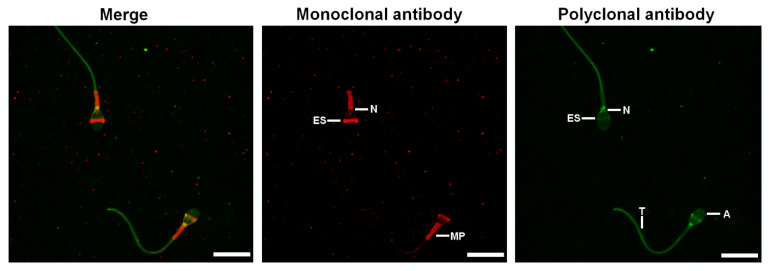
Double immunofluorescence with the two anti-HSP70 antibodies. Purified human spermatozoa were incubated in a capacitating medium for 4 h. They were then fixed with 4% paraformaldehyde, permeabilized with 0.3% Triton-X-100 and stained successively with polyclonal (Proteintech, 10995-1-AP, green labelling) and monoclonal (Sigma, H5147, red labelling) anti-HSP70 antibodies. Scale bar: 10 μm. Images are maximum-intensity projections (MaxIP) obtained from z stack images using Nikon NIS Elements software. A: acrosome, ES: equatorial segment, MP: mid-piece, N: neck, and T: tail.

The localization of HSP70 in spermatozoa was assessed using monoclonal antibodies (H5147, Sigma) and polyclonal antibodies (10995-1-AP, Proteintech). In both cases, the labelling obtained was identical for non-capacitated and capacitated spermatozoa regardless of whether they were permeabilized with Triton-X-100 or not (Figure 2 and Figure S2). The monoclonal antibodies labelled the equatorial segment, the neck, and the mid-piece of the spermatozoa, while the polyclonal antibodies labelled the acrosome, the equatorial segment, the neck, and the tail of the spermatozoa (Figure 2). Double-immunofluorescence images show that the labelling obtained at the level of the equatorial segment with the polyclonal antibodies is not completely co-localized with the labelling obtained with the monoclonal antibodies (Figure 3).

## 3. Discussion

Many scientific studies demonstrate that male fertility is in decline, with an alarming decrease in sperm parameters observed over the past 50 years [59,60,61,62]. To understand the molecular mechanisms involved in male infertility, global mass spectrometry-based proteomic approaches have been used by different research groups to identify key proteins linked to infertility (e.g., see [63,64,65,66,67,68,69] for reviews). However, when focusing on a same infertility disorder, for instance, asthenozoospermia or obesity, only a small number of proteins have been identified consistently in different-independent studies, often with opposite abundance variations [64,66]. This inconsistency could be attributed to differences in the investigated groups, type of samples and identification methods used. Therefore, it is advisable to use robust and sensitive methods to target specific proteins and validate large-scale proteomic studies. This is particularly true for protein isoforms, whose sequences are very similar. Here, we used Multiple-Reaction Monitoring (MRM) mass spectrometry to investigate HSP70 isoforms in the context of sperm capacitation and motility. MRM is more robust and sensitive than conventional mass spectrometry, which makes it particularly suitable for identifying highly homologous protein isoforms, as well as proteins of low abundance [44,45].

### 3.1. Identification of HSP70 Isoforms in Human Spermatozoa

Based on an alignment of all human HSP70 isoforms, tryptic peptides allowing to discriminate each isoform were selected. These peptides were then searched in a combined sperm proteome obtained in our laboratory. By doing this, we were able to identify HSPA1, HSPA1L, HSPA2, HSPA4, HSPA4L, HSPA5, HSPA8 and HSPA9 in human spermatozoa, as described in other human sperm proteomes [9,10,11,12,13,14]. On the other hand, we were not able to detect specific peptides for isoforms HSPA6, HSPA7, HSPA12A, HSPA12B, HSPA13 and HSPA14 in our proteome. Interestingly, HSPA6 has been detected in human spermatozoa in some studies [9,11,13,33] but not in others [10,12,14]. HSPA6 is a stress-induced protein which, unlike other inducible isoforms such as HSPA1A, presents an (almost) undetectable basal level of expression in most cell types. Moreover, its expression appears to be induced by more intense stress events than for HSPA1A [70,71]. That could explain the absence of detection of HSPA6 in some sperm proteomes, including the one used in the present study. As for the other undetected isoforms, their absence in our proteome as well as in most of other published human sperm proteomes [9,10,12,13] could indicate that these isoforms are present at very low levels (HSPA7, observed in [11]; HSPA13 and HSPA14, observed in [14,38] or are absent (HSPA12A and HSPA12B).

### 3.2. HSP70 Abundance and Localization during Sperm Capacitation

During their transit in the female reproductive tract, spermatozoa undergo a succession of biochemical and physiological changes, encompassed under the term “capacitation”, allowing their functional maturation required to be able to penetrate and fertilize an oocyte [72]. However, despite its huge importance, the molecular mechanisms involved in sperm capacitation are not yet fully understood [73,74]. Several studies have shown the importance of chaperone proteins in this process [75,76,77,78,79,80]. Among them, HSP70 has been the focus of several studies in different mammalian species [20,26,27,29,81,82,83,84]. However, these studies either referred to “HSP70”, neglecting the existence of multiple isoforms in this chaperone family, or focused only on one specific isoform. To the best of our knowledge, only the isoforms HSPA5 [82] and HSPA2 [20,26,27,29] were given specific attention in the context of sperm capacitation. In the present work, we studied the effect of capacitation on all the HSP70 isoforms identified in our human sperm proteome.

Our targeted MRM investigation on the eight detected HSP70 isoforms in the sperm proteome revealed that the abundance of each isoform does not vary following capacitation. These results are in accordance with those obtained by Castillo et al. (2019) [32], who compared the complete human sperm proteome before and after capacitation by isotopic peptide labelling and LC–MS/MS. These authors identified the same 8 isoforms as in our present study and, by applying strict selection criteria for peptide quantification, they selected HSPA1A, 1L, 2, 5 and 8 isoforms to perform their statistical analysis, which demonstrated that the abundance of these isoforms did not vary between non capacitated and capacitated spermatozoa [32]. On the other hand, using 2D-PAGE combined with MALDI–TOF–MS, Secciani et al. (2009) [31] showed a variation in abundance of HSPA1L and HSPA2 between ejaculated and capacitated spermatozoa. HSPA2 was found in different spots of the 2D-PAGE gel, which could reflect a fragmentation and/or post-translational modifications (PTMs) of the protein. Noteworthy, capacitation is a transient state and only a small percentage of spermatozoa is capacitated at any given time [85,86,87,88]. Therefore, it is not excluded that small variations could have been hidden by the presence of non-capacitated spermatozoa. It could be interesting, in future studies, to perform the MRM quantification on the isolated capacitated population.

Next, we investigated the cellular localization of HSP70 by immunofluorescence on capacitated and non-capacitated spermatozoa. To the best of our knowledge, only the localization of HSPA2 and HSPA5 isoforms have been studied in a capacitation context [20,26,27,29,82]. While HSPA5 was found to have a constant localization, mainly at the level of the neck, following capacitation [82,89,90], controverted results were obtained for HSPA2. Indeed, some studies demonstrated its constant intracellular localization [20,26,27] while others showed a change in localization [29] or an increase in surface expression [23] following capacitation. In the present study, we used two different antibodies to assess the localization of different isoforms. The first one, a mouse monoclonal anti-HSP70 (H5147, Sigma), recognizes at least the HSPA1 and HSPA8 isoforms (according to the data sheet available on the Sigma website). As the immunogenic sequence and the targeted epitope are not known from the company, it is not possible to know whether other isoforms are also recognized. However, the HSPA1L isoform is also probably targeted by this antibody, as conserved regions between HSPA1 and HSPA8 are common with those from HSPA1L. The second antibody, an antigen-affinity purified rabbit polyclonal anti-HSP70 (10995-1-AP, Proteintech), was produced based on the immunogenic sequence from the HSPA1 isoform. As the sequence of this isoform is very similar to other isoforms, the antibody could also recognize the isoforms HSPA1L, 2, 5, 6, 7, 8 and 9. The HSPA4 and 4L isoforms, which have been detected in our human sperm proteomes, cannot be recognized by the two antibodies used in our study.

The use of the two antibodies by immunofluorescence revealed different labeling. The mouse monoclonal antibody labelled the equatorial segment, neck and midpiece of the spermatozoa, while the rabbit polyclonal antibody, in addition to these locations, also labelled the acrosome and the tail of the spermatozoa. These results indicate that HSPA1, HSPA1L, and HSPA8 would be present in the equatorial segment and midpiece but would be absent from the acrosome and the tail of the spermatozoa. The labelling at the level of the neck by the two antibodies could be attributed to HSPA5, as has been shown by immunofluorescence in several studies using specific antibodies [14,82,89,90]. This isoform is a resident endoplasmic reticulum chaperone [91]. In spermatozoa, the endoplasmic reticulum is ejected during the later stages of spermatogenesis and thus many studies claim the absence of this organelle in mature spermatozoa [92,93]. However, a study by Cappallo-Obermann et al. 2011 [94] showed the presence of ribosomes in the neck of spermatozoa. Thus, they hypothesized that these ribosomes may originate from remnants of the rough endoplasmic reticulum. The localization of HSPA5 in the neck is thus a further argument for the presence of reticulum within mature spermatozoa. Based on our observations, HSPA2 and HSPA9 could be localized in the acrosome, equatorial segment, midpiece and tail of the spermatozoa, as already shown in other studies [20,23,26,27,29,30,95,96]. Only the use of antibodies directed against isoform specific peptides could allow to attribute the exact location to each HSP70 isoform.

The labeling obtained with the two antibodies did not differ between non-capacitated and capacitated spermatozoa, whether they were permeabilized or not. We tested the non-permeabilized condition to investigate the possible surface location of HSP70. However, in this condition, spermatozoa were labelled with PSA, which targets glycoconjugates within the acrosome, suggesting that the membranes were permeable to antibodies after paraformaldehyde fixation, as already observed by Lamas-Toranzo et al. [97] for the inner acrosomal markers PNA and IZUMO1. Our results, showing no variation of antibody labelling between non-capacitated and capacitated spermatozoa, are consistent with those obtained for HSPA5 [82] and HSPA2 [20,26,27]. However, they differ from those obtained by Huerta-Retamal et al. [29], which showed that HSPA2 had a heterogeneous localization before capacitation (post-acrosomal region/ equatorial segment/ acrosome/ tail), whereas after 4 h of capacitation the spermatozoa had a preferential labeling at the equatorial segment, with sometimes a less intense labeling at the acrosome. Noteworthy, the presence of different patterns of labelling could be misinterpreted because of the spherical morphology of the head of the spermatozoa. The use of maximum-intensity projections (MaxIP) obtained from z stack images in the present study abolished this effect.

### 3.3. HSP70 Abundance and Sperm Motility

Sperm motility is an essential prerequisite for their ability to fertilize an oocyte. Asthenozoospermia, characterized by a low percentage of motile spermatozoa, is one of the main causes of male infertility [98]. However, the molecular mechanisms involved in sperm motility are still poorly understood. As already discussed in the Introduction of the present paper, proteomic studies comparing asthenozoospermic and normozoospermic samples identified variations in the abundance of some HSP70 isoforms. However, the variation trend differed depending on the study (Table S1, [15,17,33,34,35,36,37,38]).

We investigated the abundance of the 8 HSP70 isoforms in spermatozoa purified from 20 sperm samples presenting various total and progressive sperm motilities. We showed that the abundance of HSP70 isoforms was not correlated to the percentage of motile and progressive spermatozoa measured in the raw semen samples. To analyze more precisely the results, and to take into account the percentage of normal forms in the samples, we selected some samples that fitted into well-defined categories: astheno-teratozoospermic, normozoospermic, and teratozoospermic. The abundance of HSP70 isoforms was not significantly different between these categories. Nevertheless, these results should be considered preliminary because the astheno-teratozoospermic and teratozoospermic groups contained a limited number of samples. For the next studies, it would be crucial to increase the sample size to confirm our hypothesis.

Our results differ from most published proteomic studies, which showed variations in the abundance of some HSP70 isoforms between asthenozoospermic and normozoospermic samples [15,17,34,35,36,37,38], but are in accordance with results obtained by Guo et al. [33] (Table S1). Interestingly, some of the studies highlighting differential HSP70 abundance used 2D-PAGE combined with MALDI–TOF–MS identification [17,34,37]. The observed variations could therefore be attributed to post-translational modifications (PTMs) of HSP70 isoforms, which were not investigated in the present study.

It is important to note that, to completely remove any contaminating cells, we worked on spermatozoa purified from the semen using a density gradient while most of other studies used a single 50% or 60% Percoll layer. We therefore selected the most motile spermatozoa from the different samples. However, Siva et al. (2010) [37] and Amaral et al. [15] also purified spermatozoa using a density gradient and identified variations in the abundance of HSP70 isoforms between asthenozoospermic and normozoospermic individuals (Table S1). Our results demonstrate that selected motile spermatozoa do not differ in terms of HSP70 isoform abundance between semen samples presenting different percentages of motile spermatozoa.

## 4. Materials and Methods

### 4.1. Subjects and Ethics

Human semen samples were obtained from the fertility clinic of Ambroise Paré Hospital (Mons, Belgium) from patients undergoing routine semen analysis or from voluntary donors. All experiments conducted in this study were approved by the Ethics Committee of Ambroise Paré Hospital in Mons and by the Ethics Committee of Erasme Hospital in Brussels (protocol P2017/540). The samples were obtained with the informed written consent from all subjects. Semen samples were collected by masturbation after an abstinence period of 3–7 days and routine seminal analysis was performed according to the World Health Organization (WHO) 2021 guidelines [99]. For the comparison of capacitated and non-capacitated spermatozoa, only normozoospermic samples (volume ≥ 1.4 mL, sperm concentration ≥ 16 × 10^6^/mL, and total motility ≥ 42%) were investigated. For the study on the abundance of HSP70 isoforms in relation to sperm motility, samples presenting normal volume and sperm concentration but with various levels of total and progressive motility were used (Table S2).

### 4.2. Sperm Preparation

Purification of spermatozoa from the semen samples was carried out by centrifugation at 300× *g* for 20 min at 37 °C on a discontinuous PureSperm 40/80 density gradient (Nidacon, Mölndal, Sweden) to remove seminal plasma, somatic cells, and immature and dead spermatozoa, as described in [100] and the World Health Organization (WHO) guidelines. Purified spermatozoa recovered from the bottom of the 80% PureSperm fraction were then washed with Dulbecco’s phosphate-buffered saline (DPBS). To check the purification efficiency, staining was performed before and after purification using the Diff-Quick kit (RAL Diagnostics, Martillac, France). All purified sperm samples contained <1% of potential contaminating cells. The purified spermatozoa were processed for capacitation or were flash frozen in liquid nitrogen and stored at −80 °C until use for protein extraction.

### 4.3. Sperm Incubation in Capacitation Medium

Spermatozoa (3 × 10^6^ cells/mL) were incubated for 4 h at 37 °C in an incubator containing 5% CO_2_ in a capacitation solution composed of HAM’s F-10 Nutrient Mix (Gibco, ThermoFisher Scientific, Waltham, MA, USA) supplemented with 3 mg/mL HSA (Gynemed, Lensahn, Germany) and 100 µg/mL ampicillin. Non-capacitated spermatozoa were incubated for only a few minutes under the same conditions. For each condition, an aliquot of the sperm suspension was removed and used to assess sperm vitality, motility, and capacitation as described below.

### 4.4. Assessment of Sperm Vitality

Sperm vitality was assessed using the BrightVit kit (Microptic, Barcelona, Spain). A 10 μL aliquot of each sample was mixed with 30 μL of BrightVit solution (Microptic, Barcelona, Spain). After 5 min incubation at 37 °C, 25 μL were spread and dried on microscope slides which were then mounted with the ROTI-Histokitt kit (CarlRoth, Karlsruhe, Germany). The BrightVit solution is a hypo-osmotic medium that allows the swelling of living cells. The solution is also composed of dyes including eosin that penetrates the membranes of dead cells staining them pink, while living cells remain colorless. In this study, only the hypo-osmotic swelling test (HOST) was used to determine sperm vitality and 300 spermatozoa were analyzed for each condition.

### 4.5. Assessment of Sperm Motility

Motility analysis was performed by loading 2 µL of sperm suspension in 10 µm Leja counting chamber slides (Microptic, Barcelona, Spain) maintained at 37 °C and by recording 5–10 videos (5 sec, 50 fps) corresponding to different fields of the chamber using a DFK 33UP1300 USB 3.0 color industrial camera connected to an inverted Nikon Eclipse Ts2R Microscope. The videos were analyzed using the Motility Module of the OpenCasa system [101], and the percentage of progressive spermatozoa (i.e., moving actively, either linearly or in a large circle, regardless of speed) was calculated.

### 4.6. Assessment of Sperm Capacitation

The efficiency of capacitation was assessed by phosphotyrosine analysis in Western blot, as tyrosine phosphorylation is recognized as a hallmark for sperm capacitation [54,102]. An aliquot from each condition (0.5 × 10^6^ sz) was centrifuged at 2000× *g* for 5 min at 4 °C, washed 3 times with cold sodium phosphate buffer (PBS, pH 7.4) and the pellet was flash frozen in liquid nitrogen and stored at −80 °C until use. Proteins were extracted with SDS sample buffer (50 mM Tris, 10% Glycerol, 2% SDS, 100 mM DTT, bromophenol blue, pH 6.8), heated for 5 min at 95 °C, centrifuged, and loaded on 10% SDS-PAGE gels. After electrophoresis, the proteins were transferred onto PVDF membranes (GE Healthcare) using 25 mM Tris, 192 mM glycine, 0.05% SDS, 20% methanol as transfer buffer. The membranes were washed with PBS containing 0.05% Tween 20 (PBS-T) and then blocked for 1 h in PBS-T-5% BSA. The membranes were incubated 1 h 30 at room temperature or overnight at 4 °C with mouse anti-phosphotyrosine clone 4G10 monoclonal antibodies (05-321X, Merck, New York, NY, USA) diluted 1:20,000 in PBS-T-3% BSA. After 5 washes of 5 min in PBS-T, HRP-conjugated goat anti-mouse immunoglobulins (G-21040, ThermoFisher Scientific, Waltham, MA, USA) diluted 1:50,000 in PBS-T-3% BSA were applied for 1 h. Finally, the membranes were washed again and immunoreactive bands were visualized using the ECL Western Blotting Substrate (ThermoFisher Scientific, Waltham, MA, USA) and the Fusion FX imaging system (Vilber, Marne-la-Vallée, France). The membranes were then stripped and reprobed with rabbit anti-beta tubulin (2128S, Cell Signaling, Leiden, The Netherlands) diluted 1:2000 and HRP-conjugated goat anti-rabbit immunoglobulins (32460, ThermoFisher Scientific, Waltham, MA, USA) diluted 1:500, for loading control [103].

### 4.7. HSP70 Localization by Immunofluorescence

An aliquot of 0.5 × 10^6^ spermatozoa from capacitated and non-capacitated conditions were fixed in an equal volume of 4% paraformaldehyde (PAF, Sigma-Aldrich, Burlington, MA, USA) in PBS for 15 min at room temperature. The samples were then centrifuged at 2000× *g* for 5 min. They were washed twice with 0.05 M glycine in PBS and once with PBS. Then, a total of 0.05 × 10^6^ spermatozoa were spread on 12 mm diameter glass coverslips and air-dried. The spermatozoa were then permeabilized, or not, in PBS containing 0.3% Triton^®^ X-100 for 20 min. After blocking in PBS-3% BSA for 30 min, the coverslips were incubated overnight at 4 °C with mouse monoclonal anti-HSP70 antibody (H5147, Sigma-Aldrich) or rabbit polyclonal anti-HSP70 antibody (10995-1-AP, Proteintech, Rosemont, IL, USA) diluted 1:100 in PBS-3% BSA. Controls were performed by incubating coverslips in PBS-3% BSA without primary antibodies. Following several washes with PBS, the coverslips were incubated for 1 h at room temperature with Alexa fluor 568-coupled goat anti-mouse (A11004, ThermoFisher Scientific, Waltham, MA, USA) or anti-rabbit (A11011, ThermoFisher Scientific, Waltham, MA, USA) antibodies diluted 1:100 in PBS-3% BSA. The coverslips were washed several times with PBS and acrosome labelling was performed for 30 min at room temperature in a 60 µg/mL solution of PSA-FITC (FL 1051, Vector Laboratories, Newark, CA, USA) in PBS. Finally, the coverslips were washed again with PBS and then mounted on glass slides with Prolong Gold Antifade Mountant with DAPI (P36941, Invitrogen, Waltham, MA, USA). The slides were observed using a confocal microscope Nikon TI2-E-A1RHD25. Double immuno-labelling was performed using the same protocol but with serial incubations in both primary antibodies, followed by serial incubations in Alexa fluor 568-coupled goat anti-mouse antibody (A11004, ThermoFisher Scientific, Waltham, MA, USA) and in FITC-coupled goat anti-rabbit antibody (SA00003-2, Proteintech).

### 4.8. Identification and Selection of HSP70 Isoform-Specific Peptides

The protein sequences of human HSP70 isoforms were retrieved from Uniprot (2020_03): HSPA1A (two isoforms; P0DMV8), HSPA1B (P0DMV9), HSPA1L (P34931), HSPA2 (P54652), HSPA4 (P34932), HSPA4L (O95757), HSPA5 (P11021), HSPA6 (P17066), HSPA7 (P48741), HSPA8 (two isoforms; P11142), HSPA9 (P38646), HSPA12A (O43301), HSPA12B (Q96MM6), HSPA13 (P48723), and HSPA14 (Q0VDF9). The sequences were aligned with MUSCLE in Geneious 2021.1.1 and tryptic peptides (peptide sequences resulting from C-terminal cleavage after lysines and arginines) allowing to discriminate each isoform were selected. These specific peptides were then searched in a sperm proteome obtained through a data-dependent acquisition (DDA) proteomic analysis performed on proteins extracted from spermatozoa in different conditions using a TripleTof 6600 mass spectrometer (Sciex, Framingham, MA, USA). This database was used to extract the spectral signature for the peptides identified with high confidence and highest-quality MS/MS spectra. These spectral data were used to set up the MRM method for the detected peptides (see below).

### 4.9. Protein Extraction and Trypsin Digestion

Aliquots (2.5 × 10^6^ spermatozoa) from capacitated and non-capacitated conditions were centrifuged at 2000× *g* for 5 min at 4 °C and washed 3 times with PBS. The pellets were flash frozen in liquid nitrogen and stored at −80 °C until use. These samples, as well as flash frozen purified spermatozoa from various donors, were suspended in 50 µL of cold 50 mM K_2_HPO_4_, 8 M urea, 50 mM DTT buffer (pH 8.5) and vortexed 3 times 10 s. Mechanical lysis was performed using an ultrasound probe (IKA U50 sonicator). Three cycles of sonication of 5 s at 20% amplitude were performed at 4 °C. The samples were centrifuged briefly and incubated for 1 h at room temperature. The sulfhydryl groups of the proteins were then carbamidomethylated with iodoacetamide used in a 2.25-fold excess to DTT in the dark at room temperature for 20 min. The samples were centrifuged at 13,300 rpm for 15 min at 15 °C and the proteins contained in the supernatants were precipitated in cold 80% acetone overnight at −20 °C and were recovered by centrifugation at 13,300× *g* for 20 min at 4 °C. Then, the pellets were resuspended in 20 µL of 25 mM NH_4_HCO_3_ containing 1 µg of modified porcine trypsin (Promega, Madison, Wisconsin, USA) and incubated for 20 min at 37 °C with agitation (1300 rpm). They were then incubated overnight at 37 °C without shaking. Trypsinolysis was stopped by adding formic acid to a final concentration of 0.1%. The samples were centrifuged at 13,300 rpm for 15 min and the supernatants were stored at −20 °C.

### 4.10. Multiple-Reaction Monitoring (MRM) Analysis

The MRM analyses were performed using a QTRAP 6500+ instrument (Sciex, Framingham, MA, USA) fitted with an electrospray ionization source (150 °C, 4500 V). Test runs were performed on extracted and digested sperm proteins for transition selection and MRM method optimization using the Skyline software (20.2.0.343 MacCoss Lab, Seattle, WA, USA). Five to six transitions, y or b ions, were chosen for each peptide, and at least two peptides were analyzed for each target HSP70 isoform. The same procedure was applied for mitochondrial aconitate hydratase (Q99798) and Tektin 2 (Q9UIF3), which were used as loading controls in the study on the abundance of HSP70 isoforms in relation to sperm capacitation and motility, respectively. Indeed, mitochondrial aconitate hydratase abundance was shown to be stable during capacitation [32], and Tektin 2 abundance was shown to be stable in various proteomic studies comparing normozoospermic and asthenozoospermic samples [15,33,36,38,104]. The validated transitions are listed in Tables S3 and S4. The peptide digest from each sample was separated on a C18-reversed phase column (YMC TriArt C18, 0.3 mm, 150 mm) and peptides were eluted at a flow rate of 5 µL/min using a gradient of 5–35% (*v*/*v*) acetonitrile with 0.1% formic acid over 20 min for the study on sperm capacitation, or a gradient of 10–35% (*v*/*v*) acetonitrile with 0.1% formic acid over 25 min for the study on sperm motility. MRM data were acquired in scheduled mode with two minutes retention time window and a maximum cycle time of 1.5 min. Skyline software (20.2.0.343 MacCoss Lab) was used for visual inspection of MRM data and area under the curve integration. Peak picking for each peptide was manually refined using the transition intensity ratio and retention time as leading parameters. The intensity of all transitions was summed up for each peptide. Protein abundance was obtained as the average of the Ln-transformed area under the curve of each target peptides normalized to the average of the Ln-transformed area under the curve of the loading control peptides (i.e., aconitate hydratase or Tektin 2).

### 4.11. Statistical Analyses

Statistical analyses were performed using GraphPad Prism (v9.0.0, GraphPad software). The Shapiro–Wilks test was used to assess the normal distribution of the data, and the Bartlett test was used to assess the homoscedasticity of the residuals. Paired *t*-tests or Wilcoxon tests (in case of non-normal distribution) were used to compare non-capacitated and capacitated spermatozoa. Correlation between the abundance of HSP70 isoforms and sperm motility was evaluated by Spearman’s rank correlation coefficient. One-way ANOVA or Kruskal–Wallis tests (in case of non-normal distribution) were used to compare the abundance of HSP70 isoforms between samples from astheno-teratozoospermic, normozoospermic, and teratozoospermic individuals. For percentages, all parametric tests were performed on arcsine-transformed data. Results were considered statistically significant if *p* < 0.05.

## 5. Conclusions

We used for the first time MRM mass spectrometry to analyze all HSP70 isoforms in human spermatozoa and we showed that their relative abundance was stable between non-capacitated and capacitated spermatozoa, as well as in spermatozoa purified from semen samples varying in the percentage of motile spermatozoa. Immunofluorescence using two different antibodies confirmed the stability of HSP70 isoforms during capacitation. However, our strategy did not focus on HSP70 PTMs, which have been abundantly detected in human spermatozoa (e.g., [35,105,106,107]). Further studies should be performed to identify potential difference in HSP70 PTMs during capacitation, as this event is known to be correlated to protein phosphorylation [54]. As our study focused on the most motile spermatozoa from each semen sample, in future studies, it would be interesting to apply our MRM method to investigate the abundance of HSP70 isoforms in subpopulations from a same sample differing in motility.

## Figures and Tables

**Table 1:** Comparison of sperm parameters between non-capacitated and capacitated spermatozoa.

	Non-Capacitated (%)	Capacitated (%)	*p*-Value
Progressive motility	72.96 ± 9.64	66.28 ± 7.41	0.183
Total motility	79.14 ± 6.94	75.73 ± 7.23	0.406
Vitality	90.80 ± 2.08	89.37 ± 2.09	0.043

Data are shown as the mean ± SD from N = 8 (progressive and total motility) and N = 9 (vitality) donors. Data between non-capacitated and capacitated spermatozoa were compared using a paired *t*-test.

**Table 2:** Influence of capacitation on the relative abundance of HSP70 isoforms in human spermatozoa.

UniprotKB Accession Number	HSP70 Isoform	Peptide Number ^a^	Fold Change Capacitated/Non-Capacitated ^b^	*p*-Value ^c^
P0DMV8	HSPA1A	3	1.04	0.307
P34931	HSPA1L	3	1.03	0.998
P54652	HSPA2	5	1.07	0.071
P34932	HSPA4	2	1.08	0.071
O95757	HSPA4L	3	1.03	0.267
P11021	HSPA5	4	1.08	0.019
P11142	HSPA8	2	1.09	0.140
P38646	HSPA9	2	1.07	>1

^a^ Number of isoform-specific peptides used for the MRM analysis. ^b^ Ratio of the normalized abundance of each isoform in the capacitated spermatozoa to its abundance in the non-capacitated spermatozoa. Mean for six replicates. ^c^ The abundances of each isoform in non-capacitated and capacitated spermatozoa were compared with a paired *t*-test (all isoforms except HSPA9) or a Wilcoxon matched-pairs signed rank test (HSPA9).

**Table 3:** Correlation analysis between the relative abundance of HSP70 isoforms in human spermatozoa and percentage of motile and progressive spermatozoa in the raw semen samples.

UniprotKB Accession Number	HSP70 Isoform	Peptide Number ^a^	Total Motility		Progressive Motility	
			r	*p*-Value	r	*p*-Value
P0DMV8	HSPA1A	2	−0.096	0.687	−0.026	0.915
P34931	HSPA1L	2	0.323	0.158	0.261	0.266
P54652	HSPA2	2	0.319	0.171	0.308	0.187
P34932	HSPA4	2	−0.223	0.346	−0.184	0.437
O95757	HSPA4L	2	−0.168	0.478	−0.049	0.838
P11021	HSPA5	3	−0.313	0.179	−0.251	0.287
P11142	HSPA8	2	−0.104	0.663	−0.064	0.789
P38646	HSPA9	2	0.056	0.816	0.068	0.774

^a^ Number of isoform-specific peptides used for the MRM analysis. Correlation analysis was performed using Spearman’s rank correlation coefficient.

**Table 4:** Comparison of the abundance of HSP70 isoforms between astheno-teratozoospermic, normozoospermic and teratozoospermic samples.

	Fold Change A/N	Fold Change A/T	Fold Change N/T	*p*-Value ^a^
HSPA1A	1.05	1.22	1.16	0.32
HSPA1L	0.79	0.76	0.97	0.35
HSPA2	0.86	0.91	1.07	0.63
HSPA4	1.35	1.05	0.78	0.13
HSPA4L	1.09	1.15	1.05	0.73
HSPA5	1.50	1.05	0.70	0.27
HSPA8	1.80	2.22	1.23	0.91
HSPA9	0.93	1.09	1.18	0.54

Values are the ratio of the normalized abundance of each isoform in astheno-teratozoospermic (A; N = 3), normozoospermic (N; N = 7) and teratozoospermic (T; N = 3) samples. ^a^ The abundances of each isoform in the different groups were compared with one-way ANOVA (all isoforms except HSPA1L and HSPA2) or a Kruskal–Wallis test (HSPA1L and HSPA2). As no significant differences were observed, results from the Tukey’s multiple comparisons test and Multiple Kruskal–Wallis’s test are not presented.

## Data Availability

Not applicable.

## Supplementary Materials

The following are available online at https://www.mdpi.com/article/10.3390/ijms23126497/s1.
