# Supplementary material for: Targeted Analysis of HSP70 Isoforms in Human Spermatozoa in the Context of Capacitation and Motility
Source: Int J Mol Sci. 2022 Jun 10;23(12):6497. doi: 10.3390/ijms23126497 (PMC9224233; doi:10.3390/ijms23126497)
Supplement: Supplementary file 1 [file ijms-23-06497-s001.zip › Supplementary Information.pdf]

Targeted analysis of HSP70 isoforms in human spermatozoa in the context of capacitation and motility

Sarah Grassi[1], Marie Bisconti[1], Baptiste Martinet[2], Vanessa Arcolia[3], Jean-François Simon[3], Ruddy Wattiez[4], Baptiste Leroy[4], Elise Hennebert[1*].

[1] Laboratory of Cell Biology, Research Institute for Biosciences, Research Institute for Health sciences and Technology, University of Mons, Place du Parc 23, 7000 Mons, Belgium

[2] Evolutionary Biology & Ecology, Université Libre de Bruxelles, Avenue Paul Héger - CP 160/12, 1000 Brussels, Belgium

[3] Clinique de Fertilité Régionale de Mons, CHU Ambroise Paré Hospital, Boulevard Kennedy 2, 7000 Mons, Belgium

[4] Laboratory of Proteomics and Microbiology, CISMa, Research Institute for Biosciences, University of Mons, Place du Parc 23, 7000 Mons, Belgium

***Corresponding author:**

Elise Hennebert, Laboratory of Cell Biology, Research Institute for Biosciences, Research Institute for Health sciences and Technology, University of Mons, Place du Parc 23, 7000 Mons, Belgium

E-mail address: elise.hennebert@umons.ac.be

Table S1. Variation in HSP70 isoform abundance in asthenozoospermic samples in comparison to normozoospermic samples in different studies

	HSPA1	HSPA1L	HSPA2	HSPA4	HSPA4L	HSPA5	HSPA6	HSPA8	HSPA9
Martinez-Heredia et al. 2008			↑						
Siva et al. 2010			↓						
Parte et al. 2012	↑	↑	↓			↓	↑	↑	
Amaral et al. 2014	=	=	↑	=	=	=		=	=
Hashemitabar et al. 2015			↓						↓
Saraswat et al. 2017	=	↓	↑	↓	=	=	=	↑	↑
Guo et al. 2019	=	=	=	=	=	=	=	=	=
Yang et al. 2022					↑				↓

↑ : up-regulation in asthenozoospermic samples, ↓ : down-regulation in asthenozoospermic samples, = : no variation, empty cells: isoform not detected or for which no information is available.

Table S2. Parameters of the semen samples included in the study, information from the donors, and the experiment(s) for which they were used

Donor	Age (years)	Volume (ml)	Viscosity	pH	Sperm count (x 10^6 sperm/ml)	Total motility (%)	Progressive motility (%)	Normal morphology (%)	Body mass index (BMI) (kg/m2)	Experiment[a]	Group[b]
1	29	3.5	normal	7.9	59.3	63.1	42.4	3.3	25.1	i, iii	T
2	20	6	increased	8.7	52.8	73.2	49.3	8.3	19.9	i, ii, iii	N
3	22	5.5	normal	8.1	22.5	47	31.3	5.1	18.4	iii	
4	31	2.2	normal	8.1	34.4	47.5	34.4	0.8	23.9	iii	
5	27	5.5	normal	8.1	46.8	45.1	22.7	17.5	33.3	iii	
6	21	5.2	normal	7.8	25.3	57.5	40.4	1.7	19.4	ii, iii	T
7	30	4.6	normal	7.9	92.7	52.6	28.1	4.2	38.3	iii	
8	29	1.5	normal	8.1	66.6	89.5	71.6	4	22.4	iii	N
9	31	2.3	normal	7.9	291.9	98.4	79.1	5.8	22.6	ii, iii	N
10	28	2	increased	8.5	239	85	54.1	3.1	25.9	ii, iii	T
11	41	10	normal	7.9	87	37.9	20.9	0.8	24.3	iii	A
12	20	5.5	increased	7.9	31.9	71.8	61.6	10.1	26.7	ii, iii	N
13	22	2.5	increased	7.9	17.5	27.3	18.3	<1	17.4	iii	A
14	32	4	normal	7.8	120.3	73.8	49.3	5	23.1	ii, iii	N
15	54	6.1	normal	8.1	28.5	47.7	37.8	1.7	33.2	iii	
16	42	4.7	normal	8.4	21.5	46.3	32.9	1.7	26.3	iii	
17	23	7	normal	8.1	21.2	36.9	24.3	2.4	20.8	iii	A
18	26	4	hyperviscous	7.9	95.3	71.5	41.6	0.8	22.8	iii	
19	43	9	normal	8.1	47.8	61.4	44.3	4.1	29.4	iii	N
20	36	3.5	normal	7.7	71.5	84.4	68.1	5.3	20.5	iii	N
21	30	2.8	normal	8.1	38.5	79.3	63.2	4	29.1	i	
22	29	6	normal	8.5	110.3	80.3	53.8	<1	23.4	i	

Cells highlighted in red denote parameters whose values are outside the reference values provided by the World Health Organization (WHO) 2021 guidelines.

[a] i: immunofluorescence, ii: MRM for capacitation, iii: MRM for motility

[b] Groups used for the comparison of HSP70 isoform abundance in relation to sperm motility (following parameters defined in the Results section). A: astheno-teratozoospermic samples, N: normozoospermic samples, T: teratozoospermic samples.

Table S3. Transition list for the MRM analysis for the study on the abundance of HSP70 isoforms in relation to sperm capacitation → Excel file

Table S4. Transition list for the MRM analysis for the study on the abundance of HSP70 isoforms in relation to sperm motility → Excel file

Table S5. Sperm parameters for non-capacitated and capacitated spermatozoa from the 9 individuals used for the analysis of the influence of capacitation on HSP70 abundance and localization

	Progressive motility (%)		Total motility (%)		Vitality (%)	
Donor	Non-capacitated	Capacitated	Non-capacitated	Capacitated	Non-capacitated	Capacitated
1	74.04	63.01	79.81	71.23	90.00	86.67
2	ND	57.89	ND	80.45	92.00	89.37
6	71.62	61.70	77.03	72.34	93.67	92.00
9	71.88	80.58	73.96	84.47	86.67	86.00
10	87.50	57.81	89.29	65.63	89.77	91.33
12	80.00	73.13	87.69	82.09	91.33	91.00
14	61.73	61.11	72.84	68.06	89.40	89.67
21	78.91	65.38	82.31	80.77	92.33	90.33
22	58.02	67.48	70.23	81.30	92.00	88.00

ND: not determined.

Table S6. Raw data from the MRM analysis of the study on the abundance of HSP70 isoforms in relation to sperm capacitation → Excel file

Table S7. Raw data from the MRM analysis of the study on the abundance of HSP70 isoforms in relation to sperm motility → Excel file

Table S8. Comparison of total and progressive sperm motility between astheno-teratozoospermic, normozoospermic, and teratozoospermic samples

	Astheno-teratozoosperm N=3	Normozoosperm N=7	Teratozoosperm N=3	F	p-value
Total motility (%)	34.04 ± 5.86	78.93 ± 12.50	68.53 ± 14.53	9.88	0.0043
Progressive motility (%)	21.16 ± 3.01	60.47 ± 13.17	45.63 ± 7.40	14.37	0.0011

Data are shown as mean ± SD. Data were compared using one-way ANOVA.

Table S9. Tukey's multiple comparisons of total and sperm progressive motility between astheno-teratozoospermic, normozoospermic, and teratozoospermic samples

	Mean difference	95% confidence interval of difference	p-value
Total motility			
A vs N	-0.4953	-0.8009 to -0.1897	0.0032
A vs T	-0.3619	-0.7235 to -0.0003270	0.0498
N vs T	0.1334	-0.1722 to 0.4390	0.4816
Progressive motility			
A vs N	-0.4173	-0.6312 to -0.2034	0.0009
A vs T	-0.264	-0.5171 to -0.01097	0.0412
N vs T	0.1532	-0.06064 to 0.3671	0.1716

A: astheno-teratozoospermic samples, N: normozoospermic samples, T: teratozoospermic samples.

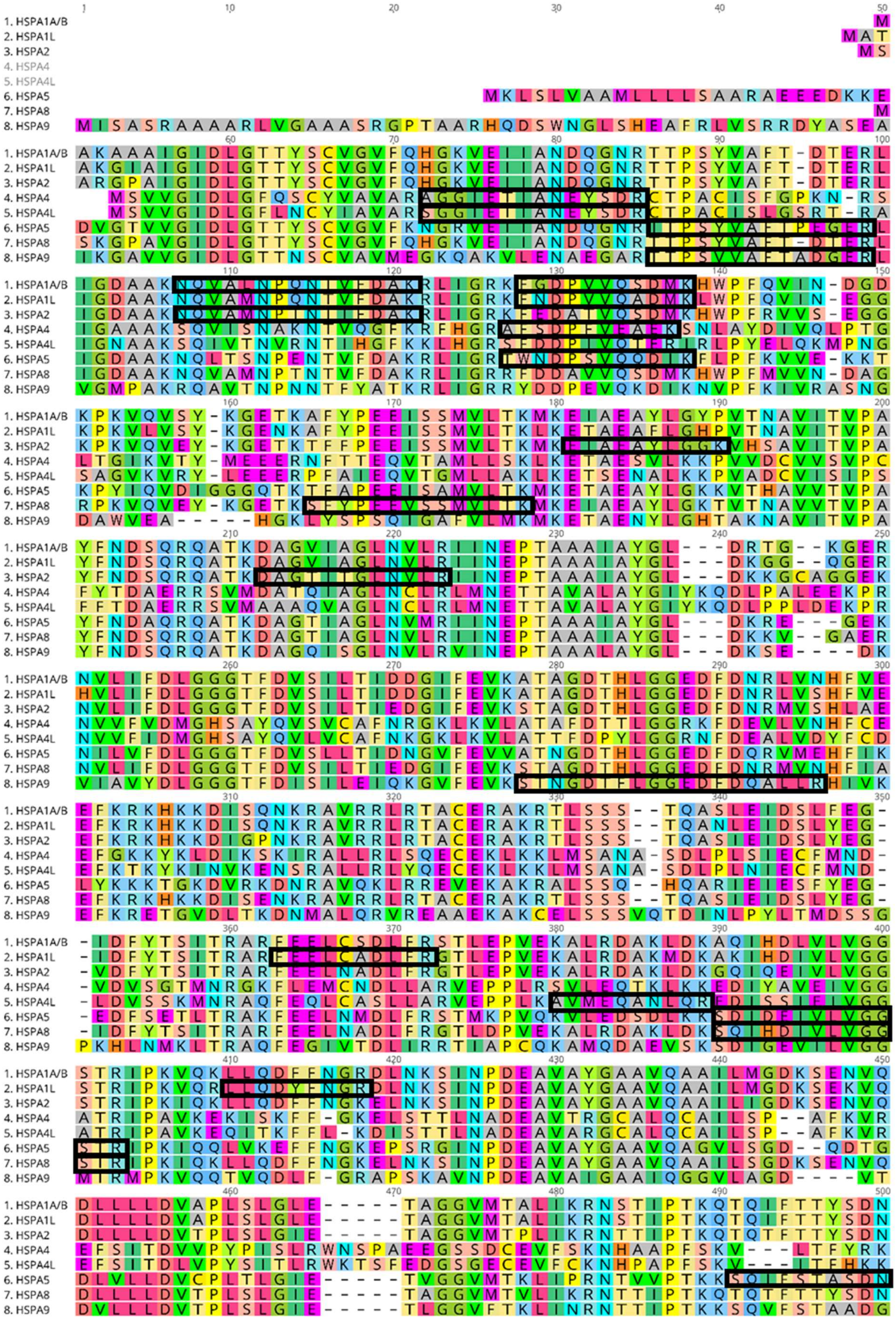

1. HSPA1A/B
2. HSPA1L
3. HSPA2
4. HSPA4
5. HSPA4L
6. HSPA5
7. HSPA8
8. HSPA9

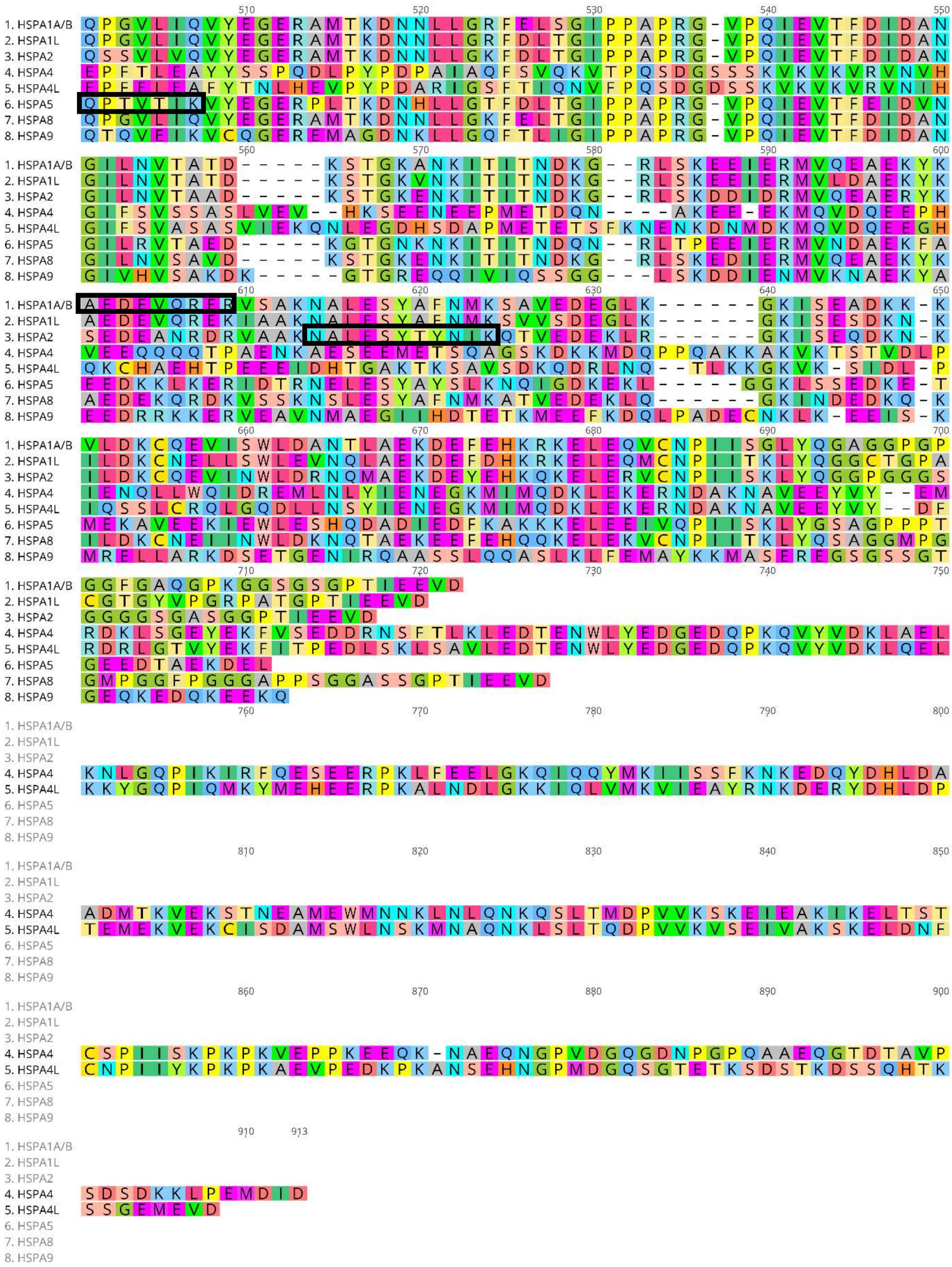

Figure S1. Alignment of the HSP70 isoforms investigated in MRM. Tryptic peptides specific from each isoform are highlighted in black rectangles.

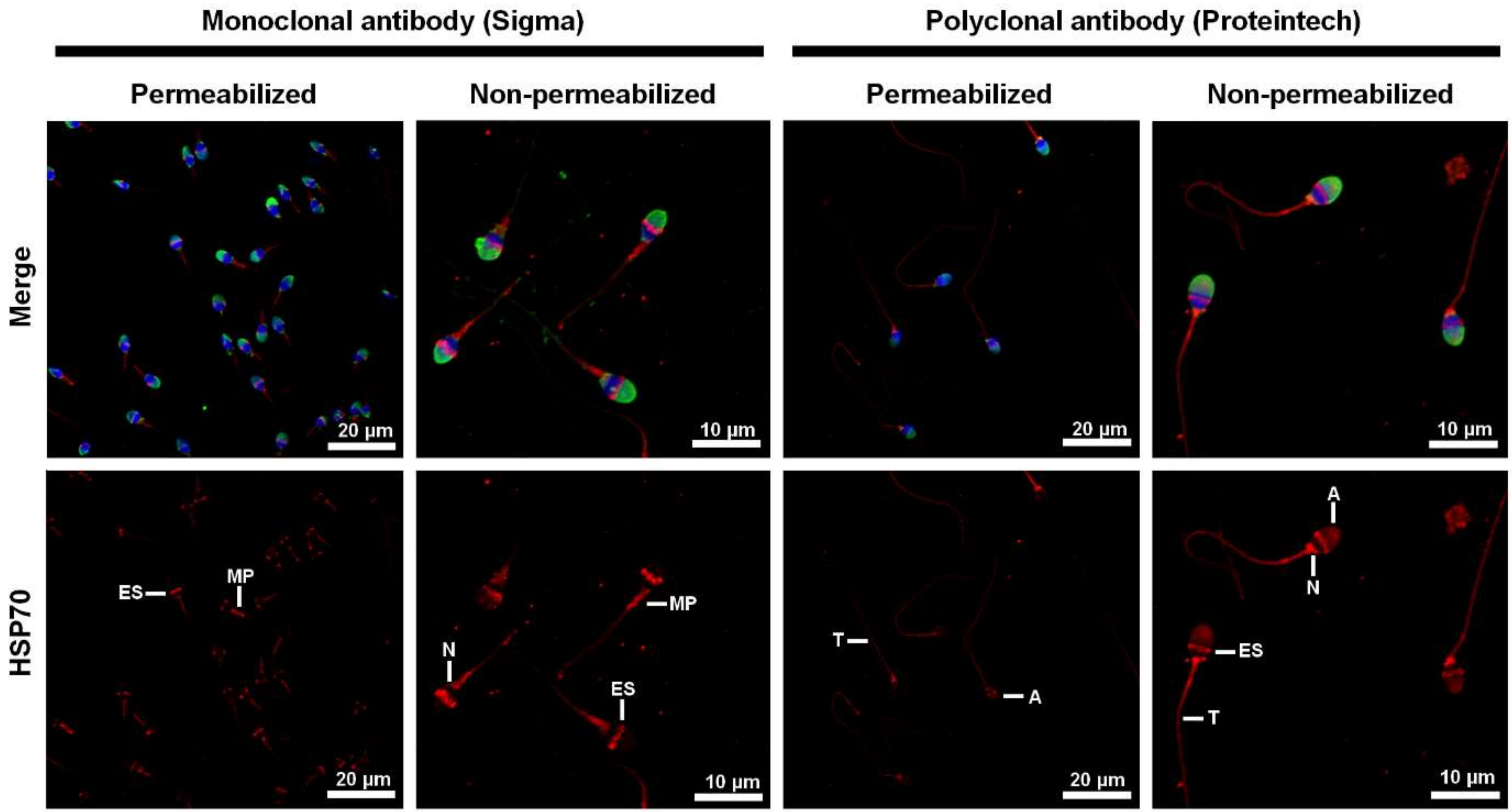

Figure S2. Localization of HSP70 in permeabilized and non-permeabilized human spermatozoa. Non-capacitated human spermatozoa were fixed with 4% paraformaldehyde, permeabilized or not with 0.3% Triton-X-100, and stained with monoclonal (Sigma, H5147) or polyclonal (Proteintech, 10995-1-AP) anti-HSP70 antibodies. Red: Hsp70, Blue: DAPI staining of the nucleus, Green: PSA-FITC staining of the acrosome. Images of the non-permeabilized conditions are maximum intensity projections (MaxIP) obtained from z stack images using Nikon NIS Elements software. Representative results of N=4 experiments. A: acrosome, ES: equatorial segment, MP: mid-piece, N: neck, T: tail.
